# Gynecology

**Published:** 1876-06

**Authors:** 


### II. Gynecology.

1. *On so-called "Ulcerations" of the Os Uteri.* Dr. Clifton E. Wing. (*Boston Med. & Surg. Journal.*)

This subject is treated with such crispness and vigor, in a paper read before the Norfolk District Medical Society, Jan. 11, 1876, that we cannot forbear making lengthy extracts from it.

What is commonly considered and treated as ulceration of the womb is not ulceration at all, but one of two conditions, both of which, once clearly understood, are simple enough. In the first condition, frequently found in its typical form in women who have not borne children, and where the cervix and os retain the normal shape, there is seen a red abrasion often entirely encircling the os; it is occasioned by the irritating discharge poured out by a uterus affected with catarrh, or, as is commonly said, endometritis. We all know how often comes a so-called cold in the head, with its accompanying discharge from the nose; the uterine mucous membrane is liable to a similar catarrhal discharge. The woman affected with a discharge from the nose removes it by the use of the handkerchief, and so prevents it from excoriating the upper lip and the edge of the nostril; if the discharge is still sufficiently irritating or excessive to cause some excoriation, perhaps the use of the handkerchief is supplemented by an application of cold cream or other unguent to the part. Now let the same woman, from, it may be, the same cause, have a uterine catarrh. She cannot keep the irritating discharge constantly removed from the surface of the cervix uteri, nor can she practically apply any unguent; and the end of the cervix in that woman is soon in the same condition as the upper lip and nostril of the little ragged boy who runs about the street on a wintry day, having no handkerchief to use and no cold cream to apply: namely, the epithelium is removed, and a raw, excoriated surface comes to view.

Catarrh of the uterus has generally become chronic before the patient applies to the physician, and the papillæ at the abraded spot, as a result of long-continued irritation, frequently have become much hypertrophied, and deceive the inexperienced eye into diagnosticating granulations. Too often, additional irritation is caused by the physician, who, totally misunderstanding the case, happens to cure the "ulcer" by lunar caustic or his other "favorite application." What educated physician would think of attacking the excoriated nostril and upper lip referred to by "burning out the 'ulcer,'" giving no attention to the catarrh, its cause? Why should we do the same thing in exactly the same condition in another part of the body? If the discharge be stopped by proper applications to the inner surface of the uterine cavity, the so-called "ulceration" will take care of itself; for you may depend upon it that if you are not more skillful than most physicians in making your application, enough will be spilled upon the excoriation outside to stimulate that part sufficiently.

The other condition mistaken for ulceration is "rupture of the cervix with eversion." It is important that this be understood correctly, as many able physicians, and specialists even, have failed entirely to recognize the real trouble or its proper treatment, and it explains those cases which have been described as "severe ulcerations which require active treatment," wherein the strongest acids and caustics, the actual cautery, and even amputation of the cervix, have been tried in misdirected attempts to bring about a return to a normal state. Dr. Emmet was the first to recognize and describe the true condition in this disease, and to invent and practice the operation for its radical cure. To him we are indebted for one of the most important steps which modern gynecology has taken in its march of improvement.

Rupture of the cervix uteri occurring in labor is generally thought to be rare, and to an extent sufficient to attract the attention of the accoucheur by symptoms is so, but it occurs in a less degree in a large proportion of childbirths. As pointed out by Dr. Emmet, if the rupture be anterior or posterior to the os, the side walls of the vagina tend to hold the torn surfaces together, so that union usually occurs: but if it takes place laterally from the os, the rupture tends to gape, the posterior lip being held back by resting against the posterior wall of the vagina, while the anterior lip shoves forward in the axis of the vagina. The condition is analogous to that of ectropium where by the rolling outward of the lid the bright-red conjunctiva is seen. Now let any physician who does not understand the condition examine with a speculum, and what does he see? Instead of the small, conical cervix which he has expected would fall readily into the end of his speculum, he finds a condition of things which startles him. With his largest speculum he cannot command a view of the whole cervix. The anterior and posterior lips, as he sees them spread out, while he thinks he is looking at their ends only, seem to him wonderfully hypertrophied. This appearance of hypertrophy, which is so nicely produced by eversion, may be demonstrated by any one by simply standing before a mirror and observing the lips in their usual position when the mouth is closed; then noticing the appearance produced by forcibly everting them, the upper lip being rolled up toward the nose and the lower lip being pulled down toward the chin; the apparent hypertrophy vanishes astonishingly when the lips are allowed to regain their normal position. The cervix soon becomes so flattened out that traces of a rupture are not visible to an untrained eye, and the observer sees what he has no doubt is the external os, but what in reality is the opening of the canal at the level of the upper limit of the rupture. All about this opening he sees a large extent of red, raw-looking surface, nothing more nor less than the mucous membrane of the canal rolled outward as above explained, its bright color contrasting with that of the vagina and external surface of the cervix almost as markedly as does the color of the mucous membrane of the mouth with that of the skin ; but as the external os is thought to be before the eye, the diagnosis of a hypertrophied cervix with ulcerations seems to be clear. Let any one evert the lips before a mirror as noted above, and see how readily the healthy red mucous membrane, with its little irregularities of surface might appear, to one *expecting* to see inflammation and ulceration, to be in such condition, and it is easy to understand how the physician who all his life has heard of ulcerations of the uterus is liable to be misled.

Moreover, this eversion of the mucous membrane is apt often enough to give rise to a catarrhal discharge from the canal, if this is not present already. Such a discharge will produce excoriation here more readily than when the cervix is in its normal shape. In the everted mucous membrane the glandulæ nabothi frequently become enlarged, and degenerate into cysts, some on the surface, others deeper ; this is another condition which is mistaken for granulations.

The rupture may take place on one side only, and be small in extent, yet cause much rolling out. In old cases all traces of it are effaced by the flattening out of the cervix on the posterior wall of the vagina, and when seen, perhaps the mucous membrane, by the formation and bursting of the little cysts, or it may be by the applications it has received, has become hard and fibrous. The amount of eversion depends much upon the extent of the rupture at the external os. A rupture which is limited here but extends upward along the uterine canal will not cause much eversion, but will give an open, patulous canal, a condition often met with, and puzzling to one who does not understand its cause.

The only safe rule to follow is this: In case of a large cervix always examine to see if eversion be not present. In hypertrophy the cervix enlarges gradually towards the body ; but, as is pointed out by Dr. Emmet, with eversion there is more or less of a neck above the everted parts, an approach to the shape of an inverted mushroom. The *practiced* finger can detect this. Try with tenacula, one on each lip, and see if they cannot be rolled inward ; if there be eversion, and this be done skillfully, it will surprise any one seeing the operation for the first time to note how the great mass resolves itself into the typical conical cervix, and how on restoration of the mucous membrane to its normal place the "ulceration" disappears. Sims' speculum is here essential ; the old round specula do not give room for the manipulation, and the valve specula, by distending the vagina with their branches, tend to hold the uterine lips apart.

Emmet's operation for the restoration of the cervix consists simply in refreshing the surfaces and bringing the parts together again, retaining them there by sutures until union has taken place. Of course, care is taken to leave the uterine canal intact. The operation is best done under ether, and the patient should lie quietly in bed for several days after the sutures are removed.

I think there has been more unintentional malpractice by physicians of the better class in such cases than in any other affection of the body. A short time ago I saw a patient who had been treated by a physician of good standing. He at first made applications of lunar caustic once a week; the "ulceration" proving rebellious, he came oftener and oftener, until the woman had a daily application for some weeks. The whole cervix was raw, bleeding on the slightest touch, while the physician was "fearful it was going to prove cancer." Dr. Emmet reports a similar case, and they are not very uncommon, for cancer is quite often suspected with this condition of eversion. With regard to applications, Dr. Emmet remarks: "The nitrate of silver in the solid form is in more common use, from its supposed mild action, than any other agent, yet from indiscriminate and too frequent use it has done more harm than any of the stronger caustics. It is not that I would so much deprecate its use in the hands of an expert, but, from its convenient form, it is too great a temptation for many who are the most ignorant to flatter themselves that they have mastered the art as a specialty when once in possession of a porte-caustique and speculum, This practice has become a scandal to the profession."* The stronger caustics in ignorant hands are of course worse. I have seen two cases within a year wherein there were cicatricial bands binding the cervix firmly to the vaginal wall and causing displacement, for which little could be done, and in both instances the only probable cause was the application of some caustic which had run down upon the vagina.

I would utter a caution against accepting as conclusive the statements, supported often by a report of cases, which now and then appear in the journals, that the most powerful caustics can be applied to the uterine canal up to the very fundus with wonderfully successful results and without danger. It should be remembered that the cases where this has been tried with disastrous effects never get into print. Many times when the operator thinks he has gone to the fundus he has not passed the inner os, as I have verified by observation.

As regards the choice of remedies I cannot do better than quote Dr. Emmet again: "In our selection we must use those calculated to do the least harm to the portion of the mucous membrane which may still be in a normal condition. Rare indeed is the necessity of applying, within the uterine canal, caustics, the cautery, or the strong mineral acids. It is true that these remedies act promptly, so far as to heal an erosion and to check all uterine discharge. But we cannot restore the

* Emmet, Philosophy of Uterine Disease, New York Medical Journal, July, 1874.

patient to health by so far changing the character of the mucous membrane as to leave it a mere cicatricial surface. Our ultimate success will be directly in proportion to the condition in which we leave this membrane, for we will need its healthy action in the after-treatment of the case. That individual cases escape with but little damage is only due to protection afforded by the secretions ; yet the practice, as a rule, is disastrous enough to deprecate their use."*

Let me here point out a common error. A physician has a patient who has symptoms which he does not exactly understand, and he finally concludes to make a speculum examination ; finding the cervix in the condition which we have been considering, and which he has always looked upon as ulceration, he fails to look further, thinking that he sees enough to account for all the suffering, when that which he has found may of itself be of no importance whatever. In this way I have known the worst affections of the uterus, the rectum, and the bladder overlooked. We all know of cases in which symptoms arising from disease of the most distant parts of the body have been foolishly referred to the same cause.

While decrying the ignorance which has so often led to such practice, let us not slight uterine affections. Many a uterine disease which would yield readily to treatment is neglected or not suspected, and the patient is allowed to fall unnecessarily into invalidism.

The close connection between certain cases of mental trouble and uterine disease cannot be overlooked with impunity. I do not refer to hysteria, which all know is commonly enough so associated, but to more serious affections—cases of melancholia, monomania, and the like. I have seen such patients get well at once, when seclusion in an asylum was apparently near at hand, by simply removing the source of irritation, some uterine disease. We must try to avoid both overestimating and undervaluing the importance of uterine affections.

* Emmet, Surgery of the Cervix. New York. 1869.

